# A milestone for movement ecology research

**DOI:** 10.1186/2051-3933-1-1

**Published:** 2013-07-03

**Authors:** Ran Nathan, Luca Giuggioli

**Affiliations:** Movement Ecology Laboratory, Department of Ecology, Evolution and Behavior, Alexander Silberman Institute of Life Sciences, The Hebrew University of Jerusalem, Edmond J. Safra Campus, Jerusalem, 91904 Israel; Department of Engineering Mathematics and School of Biological Sciences, Bristol Centre for Complexity Sciences, University of Bristol, Bristol, BS8 1TR UK

Movement characterizes our world in a fundamental and comprehensive manner, encompassing living and non-living entities that move in numerous ways. In particular, the movement of living organisms is incredibly frequent and diverse, taking a central part in many ecological and evolutionary processes that have shaped life on Earth. It is therefore not surprising that the scientific study of the movement of organisms is so rich, insightful and long-lasting [1–3]. In light of this favourable background, recent advances in quantifying and analyzing movements of organisms, their environments and internal (e.g. physiological and neurological) states [4–8], have led to a drastic upsurge of research on how, why, when and where animals, plants and microorganisms move. Over the last decade this surge has been interlinked with a quest to develop a general theory of organismal movements by means of new conceptual, methodological and empirical frameworks facilitating the desired integration [9–14]. This emerging transdisciplinary scientific paradigm was termed “movement ecology”, emphasizing the need to understand the movement of living organisms of all kinds in the context of their internal states, traits, constraints, and interactions among themselves and with the environment [9].

Progress in science is best achieved by a dynamic dialogue between new ideas and tools [15], often facilitated by transdisciplinary efforts putting together concepts, skills and tools developed in different disciplines to advance a new way of thinking on complex challenges [16] such as the conceptual integration of highly diverse movement phenomena. The movement ecology framework was proposed in this spirit [9], and the *Movement Ecology* journal is envisioned to endorse and bolster such a transdisciplinary approach. We thus invite innovative contributions to movement ecology research from a diverse range of specialists – ranging from ecologists and other biologists through mathematicians and physicists to computer scientists and engineers – and especially from transdisciplinary teams. Our transdisciplinary approach is reflected in the breath of the topics we wish to cover (e.g., cognitive sciences, climate change, epidemiology, population genetics, evolutionary biology and theoretical ecology; for more details, see the journal’s webpage), without limitation to specific methodologies, ecosystems, geographical regions, taxonomic groups, life forms or movement phenomena. It is also manifested in the breath and excellence of our editorial board, combining leading scientists from 18 different countries with diverse expertise and at different stages of their academic career. The common motivation of our team is to contribute to our understanding of the patterns, mechanisms, causes and consequences of movements of organisms. Accordingly, we invite manuscripts describing innovative insights into important questions in movement ecology research, and call our authors to put their findings within the broad perspective of movement ecology research.

Efficient communication is a crucial necessity for ambitious transdisciplinary efforts to succeed [16]. Indeed, movement ecology research has recently been advanced by many workshops and meetings putting together scholars of different expertise, as well as research centres such as CAnMove (Lund University, Sweden) and Minerva Center for Movement Ecology (The Hebrew University of Jerusalem, Israel), and large projects such as ICARUS and MoveBank of Max Planck Institute of Ornithology (Germany). We consider the establishment of the new *Movement Ecology* journal an important milestone within a series of stepping-stones facilitating the progress towards refining the key concepts and building blocks of movement ecology research. The open-access publication policy of BioMed Central is highly beneficial for this purpose, making all articles freely and universally accessible online, fostering rigorous, constructive and efficient peer-review process, rapid publication in a continuous mode immediately after acceptance, archival in a number of freely accessible full text repositories, higher rates of download and citations than equivalent subscription-based journals, and hence higher visibility and scientific impact.

A member of our editorial board with relevant expertise is assigned to each submitted article, leading the peer-review process all the way from the first assessment, through the selection of reviewers to the evaluation of their reports, guiding the authors on how to improve their manuscript towards publication of innovative and important contributions in *Movement Ecology*. Authors are expected to carefully prepare their manuscript to include an informative introduction to the theoretical and empirical background, a clear definition of the research questions, objectives and hypotheses, a sufficiently detailed description of the methods allowing replication, and clear and concise illustrations of the main results and (if relevant) their statistical significance. These should be followed by a discussion summarizing the main findings, putting them in context of previous findings and broader movement ecology concepts (e.g. comparison to different organisms, different environments, different movement phenomena), as well discussion of the main pros and cons of the research approach, with concluding remarks on key implications and future research directions. We aim for a rapid submission-to-first-decision cycle, and require proficient and rapid handling from our reviewers, editors and editorial office. We invite research articles (both theoretical and empirical), reviews and perspectives of major and emerging topics, meeting reports, and database and methodological articles—all should focus on the movement of the whole organism, across any spatial and temporal scale, organizational level and theme. We anticipate a growing flow of independent submissions, alongside solicited reviews and perspectives, and Special Features resulting from designated workshops and symposia.

Articles published at the launch of *Movement Ecology* provide a flavour of the broad scope designated for this journal. Lyons et al. [17] developed an algorithm incorporating time in movement-based home range estimation methods, and illustrated its application for springbok dataset from Namibia. Dodge et al. [18] introduced the Env-DATA path annotation system of MoveBank enabling researchers to couple tracks of any movement type with data on a plethora of environmental variables matching each individual track. This long-desired tool would facilitate a comprehensive assessment of the basic question how movements are shaped by the environment. Safi et al. [19] used this environmental path annotation of MoveBank to assess wind effects on flight of migrating birds of 8 waterfowl species, highlighting the requirement to quantify bird and wind conditions in short time intervals due to scale-dependent biases. Other manuscripts submitted to date to *Movement Ecology* that have not yet completed a full peer-review process have applied a variety of research approaches to investigate many other aspects of movement ecology across diverse taxonomic groups, spatiotemporal scales and levels of organization.

Finally, on behalf of the whole journal team – the publisher, the editors-in-chief, the editorial board members and the editorial office – we would like to reemphasize our commitment to provide a proficient, constructive and efficient peer-review and editorial work, to accomplish the important milestone of making *Movement Ecology* the primary forum for innovative and influential publications to better understand the movements of organisms. Yet, having a successful journal is a community goal, requiring, first and foremost, that you – the large and rapidly increasing community of movement ecologists – will indeed prioritize submission of your most innovative and exciting movement ecology work to this new journal.
